# Supplementary material for: Identification of photoperiod-induced specific miRNAs in the adrenal glands of Sunite sheep (Ovis aries)
Source: Front Vet Sci. 2022 Jul 22;9:888207. doi: 10.3389/fvets.2022.888207 (PMC9354845; doi:10.3389/fvets.2022.888207)
Supplement: Supplementary file 1 [file Data_Sheet_1.ZIP › Supplementary file/miRDeep2 file.zip!/miRDeep2 file/Know_Structure/SPLP42A2/oar-mir-10b.pdf]

miRBase precursor : oar-mir-10b
Total read count : 745034
oar-miR-10b read count : 741855
remaining reads : 3179

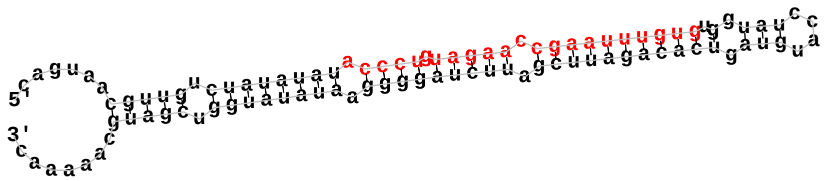

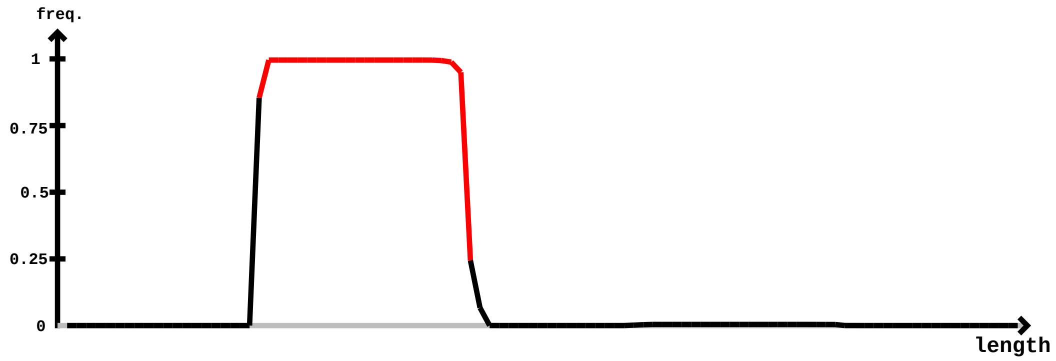

oar-miR-10b

5'- caguaacguugucuauauauacccuguagaaccgaauuugugugguauccauguagucacagauucgauucuaggggaauauauggucgaugcaaaaac -3' exp

......(((((.((((((((.((((.(((((.((((((((((.(.(((....))).)))))))))))).))))))))).)))))))).)))))).......	reads	mm	sample
caguaacguugucuauaua..	1	0	seq
..................auacccuguagaaccgaauuug...	3	0	seq
..................auacccuguagaaccgaauuugu..	15	0	seq
..................auacccuguagaaccgaauuugug...	2	0	seq
...................uacccuguagaaccgaa...	18	0	seq
...................uacccuguagaaccgaau..	154	0	seq
...................uacccuguagaaccgaauu...	1326	0	seq
...................uacccuguagaaccgaauuu..	3636	0	seq
...................uacccuguagaaccgaauuug...	26046	0	seq
...................uacccuguagaaccgaauuugu..	490477	0	seq
...................uacccuguagaaccgaauuugug...	94886	0	seq
...................uacccuguagaaccgaauuugugu..	19990	0	seq
...................uacccuguagaaccgaauuugugug...	26	0	seq
...................uacccuguagaaccgaauuugugugg..	2	0	seq
...................uacccuguagaaccgaauuuguguggu...	1	0	seq
....................acccuguagaaccgaau..	12	0	seq
....................acccuguagaaccgaauu...	107	0	seq
....................acccuguagaaccgaauuu..	477	0	seq
....................acccuguagaaccgaauuug...	2348	0	seq
....................acccuguagaaccgaauuugu..	35065	0	seq
....................acccuguagaaccgaauuugug...	37487	0	seq
....................acccuguagaaccgaauuugugu..	29589	0	seq
....................acccuguagaaccgaauuugugug...	102	0	seq
....................acccuguagaaccgaauuugugugg..	5	0	seq
....................acccuguagaaccgaauuuguguggu...	1	0	seq
.....................cccuguagaaccgaauu...	1	0	seq
.....................cccuguagaaccgaauuu..	1	0	seq
.....................cccuguagaaccgaauuugu..	7	0	seq
.....................cccuguagaaccgaauuugug...	9	0	seq
.....................cccuguagaaccgaauuugugu..	36	0	seq
......................ccuguagaaccgaauuugu..	6	0	seq
......................ccuguagaaccgaauuugug...	8	0	seq
......................ccuguagaaccgaauuugugu..	7	0	seq
.......................cuguagaaccgaauuugu..	1	0	seq
.......................cuguagaaccgaauuugug...	3	0	seq

oar-miR-10b

```
caguaacguugucuauauauacccuguagaaccgaauuugugugguauccauguagucacagauucgauucuaggggaauauauggucgaugcaaaaac
.......................cuguagaaccgaauuugugu........................................................   1      0   seq
........................................................ucacagauucgauucuaggggaau...................   1      0   seq
.........................................................cacagauucgauucuaggggaa....................   6      0   seq
.........................................................cacagauucgauucuaggggaau...................   11     0   seq
..........................................................acagauucgauucuagggg......................   4      0   seq
..........................................................acagauucgauucuagggga.....................   40     0   seq
..........................................................acagauucgauucuaggggaa....................   49     0   seq
..........................................................acagauucgauucuaggggaau...................   1041   0   seq
..........................................................acagauucgauucuaggggaaua..................   3      0   seq
..........................................................acagauucgauucuaggggaauau.................   1      0   seq
...........................................................cagauucgauucuagggg......................   5      0   seq
...........................................................cagauucgauucuaggggа.....................   36     0   seq
...........................................................cagauucgauucuaggggaa....................   31     0   seq
...........................................................cagauucgauucuaggggaau...................   1182   0   seq
...........................................................cagauucgauucuaggggaaua..................   12     0   seq
...........................................................cagauucgauucuaggggaauau.................   7      0   seq
...........................................................cagauucgauucuaggggaauaua................   1      0   seq
............................................................agauucgauucuagggg......................   3      0   seq
............................................................agauucgauucuaggggа.....................   12     0   seq
............................................................agauucgauucuaggggG.....................   2      1   seq
............................................................agauucgauucuaggggaa....................   13     0   seq
............................................................agauucgauucuaggggaau...................   660    0   seq
............................................................agauucgauucuaggggaaua..................   23     0   seq
............................................................agauucgauucuaggggaauau.................   32     0   seq
............................................................agauucgauucuaggggaauaua................   3      0   seq
```
